# Assessment of population satisfaction with medical care in conflict conditions

**DOI:** 10.25122/jml-2023-0193

**Published:** 2024-01

**Authors:** Anzhela Biduchak, Zhanetta Chornenka, Nataliya Hopko, Mohammad Wathek Obed Alsalama, Tatiana Domanchuk

**Affiliations:** 1 Department of Social Medicine and Health Organization, Bukovinian State Medical University, Chernivtsi, Ukraine; 2 Department of Internal Medicine, Physical Rehabilitation, Sports Medicine, Bukovinian State Medical University, Chernivtsi, Ukraine

**Keywords:** patient satisfaction, medical care, conflict, primary care

## Abstract

In the context of health care reform, the primary task is to ensure the delivery of high-quality medical services and good end results in the performance of individual physicians, structural units, and general medical services. The healthcare sector is one of the most socially significant spheres of functioning in every country. The problem of conflicts presents special social importance in this field, as a result of the rather close relationship between doctor and patient. The main objective of this study was to determine patients' satisfaction with the quality of healthcare at the primary level. The survey was conducted using an electronic questionnaire. The sample consisted of 1,146 residents of Chernivtsi and the Chernivtsi region, aged 18-56 and older. Almost half of the respondents (42.5%) offered a neutral overall rating of the quality of medical services at the primary care level. Only 25.5% gave a positive valuation of the quality of health care services they received, while 32% gave a negative evaluation. Patients' actions, opinions, and ideas shape and complement industry policies and the way they are implemented. In this context, if a dialogue is established among the main actors in the healthcare system, improvements in the system can be achieved, which will lead to better health and quality of life for people in the future.

## INTRODUCTION

The World Health Organization (WHO) defines the primary healthcare unit as the main tool to solve the ‘health for all’ challenge and the foundation of the entire health system; the central figure of the primary healthcare unit is the general practitioner, otherwise known as family physician [1-2]. In October 2017, a medical reform was launched in Ukraine, which, under the Law of Ukraine No. 6327, guarantees full payment from the state budget for the necessary medical services and medicines related to the provision of emergency medical care, primary, secondary (specialized), tertiary care, palliative medical care, medical rehabilitation, medical care for children under the age of 16, as well as medical care for pregnancy and childbirth [3]. According to the reform, any medical institution (physicians, state-owned enterprises, communal non-profit enterprises, private medical institutions) that has entered into an agreement for the provision of primary medical care with the National Health Service can provide free services through family physicians, therapists, and pediatricians. Every Ukrainian who has signed a contract with a family physician in any medical institution (public or private) can receive a variety of basic services free of charge, from blood pressure evaluations to prescriptions for budget medicines. The package also includes:

1. Medical examinations;
2. Primary analyses;
3. Referral to consultations and examinations;
4. Electronic recipes;
5. Electronic sick leave;
6. Direction for medical-social expert commission;
7. Adult and pediatric palliative care.

In April 2018, the first phase of the reform started with a declaration between the patient and their family physician, allowing the patient to proactively choose their own doctor [4]. The process of autonomy of health care institutions has begun with a change in their management principles through the transformation of communal budget institutions into communal non-commercial enterprises. This laid the foundation for improving transparency and accountability, and therefore for overcoming corruption in the form of hidden payments from patients and the irrational use of investment resources and working capital. The healthcare system of Ukraine has suffered a devastating impact as a result of the Great War started by Russia in 2022. The Ukrainian healthcare system has faced severe consequences as a result of the commencement of the major conflict. This conflict has led to widespread displacement of the population, worsening of serious illnesses, the spread of rare diseases, mental health issues, burnout and displacement of healthcare workers, destruction of infrastructure and supply chains, and a significant reduction in public and government revenues. According to the WHO [5], as of the end of February 2023, there were still over 18 million people in the country, with 11,756 injured and 7,199 fatalities due to the hostilities. Additionally, 769 attacks on medical facilities were documented. While the war presents an opportunity to reform the healthcare industry and cause a ‘leap forward’ for the healthcare system, there are potential threats that need to be considered. These include the long-term impact of the conflict on the economy, such as unemployment and devaluation of the national currency (hryvnia), limited human resources, reliance on international donors and non-governmental organizations, disparities in healthcare worker salaries, inequities in the provision of healthcare services across the country, and a potential decline in trust in the government in the future. Total expenditures on the medical guarantee program in 2021 (before the war) amounted to 2.4% of gross domestic product (GDP). At the same time, a high proportion of patients' out-of-pocket expenses remained –, namely 49% of current healthcare expenses. Before the start of the war in Ukraine, healthcare expenditure was planned to be 5% of GDP. However, due to the war, the National Health Service received only 2.2% of GDP, although new war-related challenges appeared in the healthcare system. The lack of funds in led to the impossibility of completing the capital repairs of medical institutions, among others.

In a post-Soviet society, patient satisfaction (whether with the healthcare system or with healthcare in general) is a subjective assessment that is often not accounted for in the development of healthcare policy. However, identifying the percentage of patients who are satisfied with their medical care and monitoring the dynamics of this indicator over time is one of the guiding principles that can stimulate the responsiveness of the healthcare system to the needs of patients. Satisfaction with healthcare has become an essential element in evaluating the performance of healthcare systems in the European Union and other countries such as Canada, the United States, and Australia [6]. Satisfaction can be considered as a concept that indicates the connection of the healthcare system with the needs of its residents and patients and is considered an important (but not the main) factor in assessing the quality of medical care [7].

In the U.S., Canada, Australia, Norway, and other countries, evaluations of satisfaction with healthcare services became important in the 1950s and 1960s, when healthcare took on the characteristics of a market, and the patient began to be regarded a consumer: a more active subject, responsible for his own health and in charge of making decisions about diagnosis and treatment [8-10]. Therefore, feedback in the form of consumers' opinions, expectations, preferences, choices, and satisfaction with services has become very important. Some authors claim that client expectations and values should only be considered when evaluating a healthcare provider's work [11].

In the standard of measuring the quality of medical care in the population, patients' own subjective evaluation of various aspects of medical care plays an important role [12]. However, the problem of conflicts, as a result of the close relationship between the doctor and the patient, acquires special social significance in this field. For this purpose, a sociological study was conducted on residents of Chernivtsi and the Chernivtsi region aged 18-65. Participants were asked to analyze the pros and cons of the healthcare services they received, to rate these services, and to express their views on the potential for primary healthcare facilities to improve services for the population.

Conflict arises when one of the parties changes its values, resulting in a worsening of relations. Conflict can be latent if the existing situation suits all involved or interested parties, or if these parties do not yet have sufficient influence on the development of the situation. The ability to communicate with patients is one of the most valuable qualities of a doctor of any medical specialty and determines the effectiveness of the treatment and diagnosis, the degree of patient satisfaction with the medical care, as well as the assessment of the specialist's professionalism.

The concept of satisfaction with the healthcare system and medical services for patients is included in the study Health Index. Ukraine’, which is intended to compare the healthcare systems of different countries [12] or evaluate the system of one country over a long time [13]. Worldwide, the research on consumer satisfaction with medical services (e.g., patient satisfaction with primary care consultations, and satisfaction of patients with various diagnoses) is increasing yearly [14-17]. However, in post-Soviet countries, patient satisfaction is often not considered when developing healthcare policy, so it is not sufficiently researched. Therefore, the implementation of appropriate measures that would contribute to greater patient satisfaction is lacking. However, rare empirical findings show that in the world, post-communist countries show the lowest level of satisfaction with both their lives and the healthcare systems [18]. Also, the most signaled issues in medical care are long queues, lack of medicine, bribes, and disrespect from the doctor, while attributes such as lack of cleanliness and "frequent unjustified absence of a doctor at the workplace" – are mentioned to a lesser extent [19].

## Material and Methods

The main objective of the study was to determine the degree of patient satisfaction with the quality of primary medical care in conflict conditions. The sample included 1,146 people aged 18–56 and older, residents of Chernivtsi and Chernivtsi region. The research focused on patient’s satisfaction with the healthcare system as a whole and medical care in particular. The scientific analysis was implemented using a systematic approach. The work also uses the bibliosemantic method to analyze, compare, and summarize data on systems of satisfaction with the quality of healthcare in foreign countries, and the experience of their reform.

## RESULTS

A total of 1,146 people participated in the survey, of whom 544 (47.5%) were men and 602 (52.5%) were women. The main characteristics of the respondents are shown in Table 1.

**Table 1:** Participants’ characteristics

Age/gender	Male (544, 47.5%)				Female (602, 52.5%)			
Territory of residence	City		Village		City		Village	
	*n*	%	*n*	%	*n*	%	*n*	%
18-35 years	73	25.9	49	18.7	113	34.5	37	13.5
36-55 years	112	39.7	109	41.6	119	36.3	119	43.4
56 years and older	97	34.4	104	39.7	96	29.3	118	43.1
**Total**	**282**	**100**	**262**	**100**	**328**	**100**	**274**	**100**

Out of the sample, 96.9% were registered with a family physician. Moreover, 60% of the respondents visited a doctor at the state dispensary at their place of residence, while 20% chose a doctor in a dispensary outside of their place of residence. A total of 16.2% received their healthcare in a private medical center, while 3.8% were undecided on the choice of a specialist. During the past three years, 24.6% of respondents consulted a family doctor less than once a year, 28.5% consulted a family doctor 1-2 times a year, and 41.5% consulted a doctor more than 1-2 times a year. and 5.4% visited their family doctor at least once a month.

Respondents were asked to analyze their last visit to their family physician and to rate the organization of outpatient services, the effectiveness of prescribed treatments, and their general impression of the medical services they received (Table 2).

**Table 2:** Patients’ satisfaction with the provision of medical services

Gradation	Satisfaction with the attitude of the medical staff (%)			Satisfaction with the sanitary and hygienic conditions in the medical institution (%)			Satisfaction with the results of the provision of medical care (%)		
	18-35 years	36-55 years	56 and older	18-35 years	36-55 years	56 and older	18-35 years	36-55 years	56 and older
**Very dissatisfied**	5.5	5.9	5.5	10.3	13.7	14.5	3.7	8.3	7.5
**Not satisfied**	13.6	21.6	25.3	25.7	29.2	29.9	13.6	20.3	25.8
**Partially satisfied**	50.0	42.0	44.3	45.6	37.3	36.4	47.8	42.3	42.9
**Satisfied**	28.3	27.2	19.5	14.7	16.6	14.5	30.9	24.4	18.6
**Very satisfied**	2.6	3.3	5.3	3.7	3.3	4.8	4.0	4.8	5.3

The highest percentage of patients (45.6%) were partially satisfied with the sanitary conditions of the medical facility. the attitude of the medical personnel and the results of medical care delivery. The lowest percentage of respondents (3.3%) were very satisfied and the very dissatisfied patients (13.7%) and dissatisfaction with the sanitary and hygienic conditions in the medical institution were almost two times higher (29.9%) exceeded dissatisfaction with the attitude of the medical staff and the results of treatment (Table 2).

**Figure 1:**
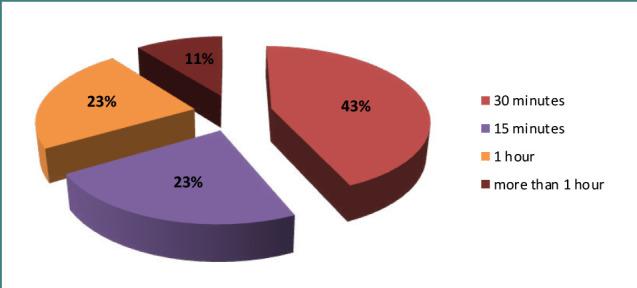
Waiting time to see a family doctor

Although at the time of the study, the reform of primary care was completed and patients could fully use the system of advanced electronic appointments with their family doctor, one of the biggest complaints of the respondents was the long waiting time to see the doctor (Figure 1).

The majority of respondents (43%) had to wait in line for 30-40 minutes, which is most likely related to the limitations of the electronic healthcare system; approximately a quarter of the respondents (23%) – waited up to 15 minutes for their turn; 23% waited in line for an hour, and 11% – for more than an hour. This is probably related to the habit of visiting a family doctor without a prior appointment.

**Figure 2:**
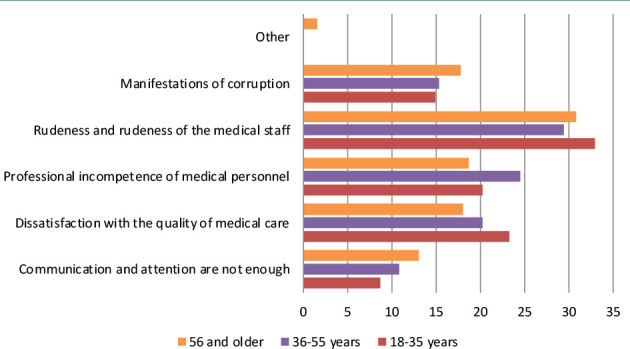
The main complaints of the respondents regarding healthcare

Furthermore, the study analyzed the existence, causes, and types of conflicts in the medical field. Today, healthcare is perceived primarily as a service provided to patients by medical institutions. Therefore, it is natural for patients to have idealized images and expectations of physicians and the treatment process when seeking consultation and treatment. Almost half of the surveyed respondents (47.3%) did not have any conflicts with medical workers, 29.1% had a conflict with medical staff, 18.7% – with doctors, and only 4.9% – with the medical institution itself. Among the main causes of conflict, patients named the following: insufficient communication and attention from medical workers, dissatisfaction with the quality of medical care, professional incompetence of medical personnel, rudeness of medical personnel, and manifestations of corruption (Figure 2).

Respondents of all age groups consider a rude negative attitude of medical staff towards patients as the most frequent cause of conflict (30.8%), followed by the professional incompetence of medical staff (21.4%), and the quality of medical care (20.2%). According to our survey and contrary to expectations, manifestations of corruption among medical personnel rank only fourth (16.1%). Lack of communication and attention from medical professionals to patients (11.0%) is in fifth place, while the lowest percentage is occupied by other causes of conflicts (0.6%). When analyzing the situations in which conflicts arise between patients and doctors, the most common situation is the deterioration of the patient’s condition at 42.6%, followed by patient duty or emergency medical assistance at 30.9%, ‘during the explanation of the treatment plan’ at 25.9%, and other circumstances at 0.6%. Conflicts in medical organizations have always existed and will continue to exist. Therefore, the issue is still relevant today. In the jurisdictional forms of human rights protection in the medical field, a distinction is made between traditional and alternative methods of dispute resolution. The traditional ones include judicial and extrajudicial. Extrajudicial is further divided into an appeal to the head of a medical institution (oral and written); an appeal to the health management body; assistance of independent public organizations and professional associations; a complaint to the prosecutor's office, and others [20]. It can be more appropriate to use alternative methods of dispute resolution, which are quite popular and widely used in Europe, the USA, Canada, Australia, and other countries. In Ukraine, alternative methods of dispute resolution are just beginning to gain popularity, based on foreign experience [21]. While there are many alternative dispute resolution methods, we are interested here in those applicable to medico-legal relationships, namely:

1.negotiation – where the parties resolve their dispute directly without the participation of others;
2.mediation – where the parties resolve their dispute with the help of an independent neutral mediator who helps them reach an agreement;
3.arbitration – where the parties resolve their dispute with the help of an independent neutral who makes binding decisions for them- an arbitrator.

In our study, a conflict with a medical worker was resolved in court only in 2.7% of cases, the remaining 97.3% of respondents resolved conflict situations in a pre-trial manner with the help of negotiations (40.8%), claims (31.5%), complaints (23.7%), and mediator (1.4%). Today, most of the interviewees prefer alternative methods of conflict resolution, because arbitration provides an opportunity for a peaceful settlement of the dispute, for the parties to reach an amicable agreement, and to maintain normal relations between the parties.

**Figure 3:**
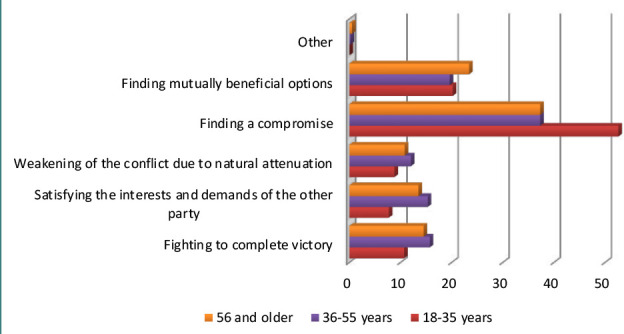
The main ways to resolve conflict in medical institutions of the Chernivtsi region

Among the most acceptable ways of resolving the consequences of the conflict (Figure 3) highlighted by respondents were fighting until complete victory, satisfying the interests and demands of the other side, weakening the conflict due to natural attenuation, finding a compromise, and finding mutually beneficial options.

**Figure 4:**
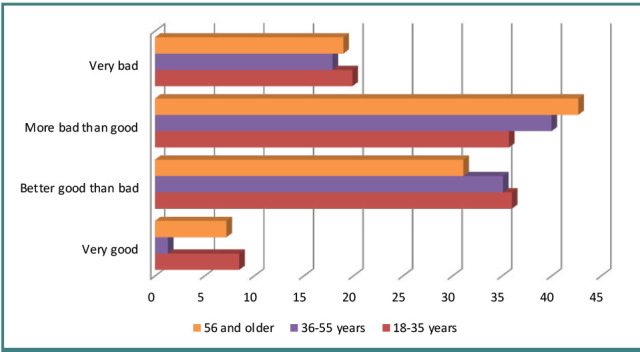
Respondents' evaluation of the efficacy of the state as the main conflict regulator between the doctor and the patient

The best ways to resolve the conflict according to the respondents are finding a compromise (40.9%), finding mutually beneficial conflict resolution options (21.1%), fighting until complete victory (14%), satisfying the interests of the opposite party – 12.8%, weakening the conflict due to natural extinction – 10.8% (Figure 3). Nowadays, patients are well aware of their rights and are not afraid to defend themselves in court. However, conflicts between patients and doctors always precede serious litigation and can eventually lead to the destruction of the reputation of the medical institution; according to Ukrainian Law No. 394/96-BP of 02.10.1996 ‘On Citizens' Actions’, the main regulator of the contentious relationship between doctors and patients today is the state (Figure 4).

The majority of respondents (40.0%) believe that the state ‘rather badly than well’ fulfills the duties of regulating conflicts between a doctor and a patient. Slightly fewer respondents (33.9%) are on the contrary confident in the power of the state to fulfill its duty, while 18.8% categorically declare that the authorities' performance of duties in doctor-patient conflict situations is ‘very bad’. The smallest number of respondents (7.5%) perceive such duties as ‘very good’, and for the most mature working age group (36-55 years old) this indicator is minimal – 1.3%.

A trend of increasing patient satisfaction with the quality of medical care at the primary level was revealed. This is in contrast to studies on satisfaction with various services which showed that Ukraine has the lowest percentage of satisfaction with public medical services among the countries of Europe and Central Asia [22]. However, the sample is not representative of the general population. Based on this, the results of the overall assessment and comparability of patients may be distorted or asymmetric. Therefore, refinements can be achieved through expert discussions and more extensive testing in the future.

## Discussion

One of the foremost imperative issues related to the estimation of satisfaction with therapeutic care is the assurance that the patient's desires are taken into consideration at the level of therapeutic administration [23-24]. Patient satisfaction can altogether impact the position of the healthcare organization within therapeutic administrations [25]. The most important outcome in studying patients’ satisfaction with therapeutic care is to improve the quality of healthcare administration. In our pursuit, we used a framework of electronic surveys for patients 18-56 years old from healthcare settings in Chernivtsi and Chernivtsi region. We used our investigation as a basis for encouraging healthcare evaluation in Ukraine, especially within conflict situations. Surveying the quality of healthcare administrations, particularly within external conflicts such as the Ukrainian war, is critical for a wide range of stakeholders, from educators, suppliers and beneficiaries of healthcare, governments, heads of therapeutic organizations, lawmakers, and citizens [26-27]. Since the care of individuals and their well-being is at the core of any healthcare framework, the wants and desires of patients ought to be a focus of the healthcare workforce [27]. Recipients of healthcare are the most important actors within this context; they have the right to high-quality therapeutic services and the right to joint decision-making during the course of their treatment [28]. Persistent fulfillment of healthcare needs should be a multidimensional pursuit; its evaluation permits the investigation of disparities between patients’ needs and the healthcare supplier's abilities.

Understanding the needs of the patients is one of the critical markers of well-being. It is progressively used in evaluating the quality of healthcare, relationships between patients and healthcare suppliers, and organizing healthcare administrations [29-30]. Blich *et al*. accept that persistent involvement measurements are the basis of deciding the responsiveness of a healthcare framework and will eventually lead to quality enhancement. The concept created by the WHO is likely to pull in more consideration, as specialists and clinics are expanding to enhance the quality of healthcare and improve security while reducing the costs of healthcare administration [31]. American analysts Dunsch *et al*. have shown the most important variables are brief waiting times, clean offices, and healthcare workers who react to the desires of patients and treat them with regard [32]. Xesfing *et al*. analyzed the impact of financial variables on satisfaction with healthcare and have shown that the proficiency of healthcare frameworks straightforwardly influences quiet fulfillment [33], which can be the premise for creating future healthcare framework assessments. In our research, satisfaction with therapeutic care was analyzed based on the conclusions of patients who were treated in primary care offices.

One of the components influencing satisfaction with therapeutic care is the waiting time. In outpatient healthcare clinics, a long waiting period, particularly between enrollment and medical consultation, leads to patient disappointment [34]. This was supported by the research of Al-Harajin *et al*. conducted in Saudi Arabia, portraying patient satisfaction related to hold-up times for healthcare visits. According to our research, nearly half of the patients had to wait more than 30-40 minutes, which had a negative effect on healthcare satisfaction. Research conducted in other nations highlights a relationship between satisfaction with the medical service and the outcome of treatment, which is additionally clarified by expert consensus and, therefore, leads to better adherence to the treatment regimen [35]. This investigation leads to a better understanding of the needs, accessibility, and quality of care in Ukraine.

For Ukraine, such things are anticipated since the wellbeing care framework has been in a state of change for a long time, and with each change of government, the concept of ‘change’ too changes. Such vulnerability leads to the truth that, within the conditions of the changed socio-political setting, the standards of healthcare and their objectives and standards of financing stay unaltered. However, each year the state progressively shifts the financing of medication to the patients - in 2015 the most noteworthy share of private investing in healthcare was recorded compared to previous years [36]. Various studies illustrate patients' exploration for elective ways to meet the requirements for therapeutic care: turning to non-traditional pharmaceuticals, self-medication, and Internet searches, among others. [37-38].

## Conclusion

Providing a comfortable environment for medical workers is an important aspect of the development of the medical field in conflict conditions, including the military, which will attract and retain qualified personnel and ensure access to quality medical care for the population. Almost half of the respondents (42.5%) gave a neutral overall assessment of the quality of the provision of medical services at the primary level, 25.5% gave a positive assessment, and 32% spoke negatively about the quality of the medical services received. To improve the quality and availability of medical care, it is also important to promote the development of private medical institutions and partnerships with charitable organizations and international agents. This will reduce the burden on the state healthcare system and provide quality services to citizens. In the post-war period, the improvement of the medical system should become a priority for the state and society in general. Humanism, accessibility, quality, and innovation must become the guiding principles in medicine for the country to recover its health and well-being after war and internal migration.

## Data Availability

The data of this study is available by request.
